# Doc

**Published:** 1894-04

**Authors:** 


"DOC."—If it has been your misfortune to be called "doc," and if this recognition has become at all general among your friends you might as well move to some other place. A man may be called a thief, a liar and a dead beat, and yet he may prosper and live upon the fat of the land. But once let him be called "doc" and his professional success is at an end. We would prefer to spend a night in the station-house, so far as its effect on our professional success is concerned rather than to have our friends notice our approach by saying, "There comes doc." If a man calls you "doc" you need never expect a penny from him for any professional services you could render. His answer is sure to be, "All right, doc, in a few days that will be all right." "Doc" means disaster. "Doc" is the culmination of all calamity. "Doc" is a catastrophe given at one stroke. "Doc" is the warning that we have reached the extreme limit of our usefulness. "Doc" is the hand which points us to the next town. Shun it, my young friend, as you would flee from a Kansas cyclone or a prairie-fire. Knock the man down who first dares speak it to you; and call upon the whole medical profession for vindication of your righteous deed. —*The National Medical Review.*

